# Quantity without numbers and numbers without quantity in the parietal cortex

**DOI:** 10.1016/j.neuroimage.2009.02.016

**Published:** 2009-06

**Authors:** Marinella Cappelletti, Neil Muggleton, Vincent Walsh

**Affiliations:** Institute of Cognitive Neuroscience and Dept of Psychology, University College London, 17 Queen Square, London WC1N 3AR, UK

## Abstract

A dominant view in numerical cognition is that processing the quantity indicated by numbers (e.g. deciding the larger between two numbers such as ‘12.07’ or ‘15.02’) relies on the intraparietal regions (IPS) of the cerebral cortex. However, it remains unclear whether the IPS could play a more general role in numerical cognition, for example in (1) quantity processing even with non-numerical stimuli (e.g. choosing the larger of ‘bikini’ and ‘coat’); and/or (2) conceptual tasks involving numbers beyond those requiring quantity processing (e.g. attributing a summer date to either ‘12.07’ or ‘15.02’).

In this study we applied fMRI-guided TMS to the left and right IPS, while independently manipulating stimulus and task. Our results showed that IPS involvement in numerical cognition is neither stimulus-specific nor specific for conceptual tasks. Thus, quantity judgments with numerical and non-numerical stimuli were equally affected by IPS-TMS, as well as a number conceptual task not requiring quantity comparisons. However, IPS-TMS showed no impairment for perceptual decisions on numbers without any conceptual processing (i.e. colour judgment), nor for conceptual decisions that did not involve quantity or number stimuli (e.g. summer object: ‘bikini’ or ‘coat’?). These results are consistent with proposals that the parietal areas are engaged in the conceptual representation of numbers but they challenge the most common view that number processing is so automatic that the simple presentation of numbers activates the IPS and a sense of magnitude. Rather, our results show that the IPS is only necessary when conceptual operations need to be explicitly oriented to numerical concepts.

## Introduction

Neuroimaging studies have shown that the parietal regions, especially those around the intraparietal sulcus (IPS)^1^ are reliably activated when processing number quantities, for instance when deciding on the larger of two numbers (e.g. Dehaene et al., 2003; Nieder, 2005). Some studies have further differentiated the fine-grained structures within the IPS and provided evidence that the bilateral horizontal segment of the IPS^2^ plays a role in quantity processing (Dehaene et al., 2003). Further evidence of the involvement of the IPS regions in number processing comes from studies investigating how this process is affected by permanent neurological damage in patients or temporary disruption following transcranial magnetic stimulation (TMS). Patients with left parietal damage, for example, can be impaired at processing number quantities (e.g. Cipolotti et al., 1991; Dehaene and Cohen, 1991; Lemer et al., 2003; Polk et al., 2001), and TMS studies have reported impaired performance in terms of increases in response times in number comparison following IPS stimulation (e.g. Andres et al., 2005; Cappelletti et al., 2007). Moreover, recent studies on developmental dyscalculia showed that this is associated with right IPS dysfunctions (Molko et al., 2003; Price et al., 2008; Rotzer et al., 2008).

Despite this converging evidence, it is still unknown whether the IPS is critical for: (1) quantity processing irrespective of the stimuli used, i.e. numerical or non-numerical; and (2) other conceptual processing of numbers that do not require quantity manipulation (e.g. attributing a summer date to either ‘12.07’ or ‘15.02’). A few studies have examined the stimulus-specific nature of quantitative processing. These have proposed that quantitative judgments on different types of stimuli may either rely on distinct subregions within the parietal lobe, or that the parietal regions are involved in a generic process of comparison of various types of stimuli. Behavioural support for the hypothesis of a generic comparison mechanism can be found in the evidence of a similar distance effect in many continua other than numerical stimuli (e.g. Fulbright et al., 2003; Johnson, 1939; Moyer, 1973). That is, the time it takes to compare two stimuli increases as the distance between the two stimuli decreases (i.e. ‘distance effect’, Moyer and Landauer, 1967). However, the results of imaging studies examining this issue are mixed: while some of these studies showed common activation of the parietal areas in quantitative judgments irrespective of the stimuli used (e.g. Fias et al., 2003), others demonstrated that different stimuli are represented in distributed and overlapping neural populations within the parietal areas (Fulbright et al., 2003; Cohen Kadosh et al., 2005; Pinel et al., 2004). These discordant results may depend on the tasks used: for instance in tasks such as number comparison, quantity information, has to be ‘extracted’ from the stimuli, whereas in the comparison of variables such as luminance and physical size (e.g. Pinel et al., 2004) or angles and lines (e.g. Fias et al., 2003), quantity information can be obtained by visual inspection of the stimuli. Thus, there is an important distinction between quantity processing based on extracting prior knowledge associated with the stimuli, and quantity processing that can be based on the physical properties of the stimulus. At present there is still a need to know whether the IPS regions are involved in quantity processing of non-numerical stimuli with similar task demands.

Although the most distinctive meaning of numbers is to express numerical quantity or cardinality (e.g. 5 items) this is not the only information that numbers convey (Wiese, 2003). They carry other types of information that do not imply quantity manipulations, for instance hours (e.g. 7.15 a.m.), dates (e.g. 2006) and mathematical constants (e.g. 3.14). Although these meanings are clearly different from quantity information, this distinction has not yet been fully explored. Suggestive evidence of this dissociation comes from lesion studies showing dissociations between quantity manipulation and processing of other non-quantity meanings of numbers (e.g. Cappelletti et al., 2008). Moreover, a recent fMRI study reported a distinction between processing numbers in arithmetical and encyclopaedic contexts, which are thought to rely on parietal and left temporal regions respectively (Lochy and Van Turennout, 2005). Some recent studies have focused on another type of non-quantity information conveyed by numbers that is order. This refers to the use of numbers to indicate the position of an item or event in an ordered sequence (e.g. the 5th items, Butterworth, 1999; Dehaene, 1997; Jacob and Nieder, 2007). A few recent studies have shown that ordinal and quantity information dissociate both behaviourally (e.g. Gevers et al., 2003; Turconi et al., 2006; Zamarian et al., 2007) and temporally: namely they take place in overlapping brain regions of the parietal cortex on different time scales (e.g. Turconi et al., 2004). All this evidence suggests a distinction between quantitative and non-quantitative meanings of numbers including ordinal information. However, it is still unknown to what extent the IPS regions are critical for these non-quantity conceptual processes of numbers, and whether the IPS is equally critical for number processes that do and do not involve quantity information.

### The present study

In this study, we applied fMRI-guided TMS and factorially manipulated stimulus (numerical and non-numerical) and task (quantity and non-quantity) independently. We aimed to test the role of left and right IPS in: (1) quantity processing with numerical or non-numerical stimuli in terms of object names; and in (2) conceptual processing of numbers requiring or not requiring the manipulation of quantity information.

To address these issues, subjects were engaged in three different categorization tasks. Two focused on the conceptual attributes of either quantity (e.g. choosing the larger of ‘12.07’ and ‘15.02’, or the larger of ‘bikini’ and ‘coat’) or category (e.g. attributing a summer date to either ‘12.07’ and ‘15.02’ or choosing a summer object between ‘bikini’ and ‘coat’). The third was a perceptual task that focused the subjects' attention on the colour of the stimulus (e.g. choosing a number or object name drawn in red) and therefore involved both attention to the stimulus and response selection as in the other tasks but without requiring a conceptual decision. We chose a non-quantity conceptual task (e.g. attributing a summer date to either ‘12.07’ or ‘15.02’) that required categorical processing of numerical information but it was unlikely to rely on quantitative manipulations.

## Material and methods

### Stimuli and tasks

The experiment was controlled using the Cogent Graphics toolbox (http://www.vislab.ucl.ac.uk/Cogent/) and Matlab7 software. The viewing distance was approximately 0.5 m. A total of 144 Arabic numbers and 144 object names were generated. Arabic numbers were presented as pairs of 3 and 4-digits separated by a dot, e.g. 12.07. They referred to a linear dimension of quantity, to dates (e.g. 12th July) or times (e.g. seven minutes past twelve in the morning). Numerals indicating quantities ranged from 1 to 31 for the first half of the numerical expression and from 01 to 59 for the second part (i.e. from 1.01 to 23.59). Numerals indicating dates were chosen to represent either summer or winter days in the Northern hemisphere; therefore summer dates included the months of June, July, and August, winter dates included the months of December, January and February. Dates were expressed in terms of day and month separated by a dot (e.g. 12th July = 12.07). Numerals referring to a date ranged from 01 to 31 for the first half of each numerical expression and from 01 to 12 for the second part (i.e. from 01.01 to 31.12). Each date indicating a summer (or winter) month was presented with either a winter (or summer) month, e.g. ‘12.07’ and ‘15.02’ or with a ‘neutral’ month, i.e. ‘12.07’ and ‘24.03’. Numerals indicating times were chosen to refer to either a sleeping or a working time approximately in terms of 8am to 6pm working day. Therefore, working times were chosen between 8am and 6pm, and sleeping times between 10pm and 7am. Times were expressed in terms of 24-hour clock with the first pair of digits referring to the hour and the other two digits, separated by a dot, referring to the minutes past the hour (e.g. 16.30 is half past four in the afternoon). Numbers referring to a time ranged from 00 to 23 for the first half of each numerical expression and from 01 to 59 for the second part (i.e. from 00.01 to 23.59). Object names referred to concrete, countable objects whose size could be unambiguously identified and that could be used in both the quantity (e.g. larger object: ‘bikini’ or ‘coat’?) and the non-quantity tasks (e.g. summer object: ‘bikini’ or ‘coat’?). In the latter task, each object name in a pair consisted of a stimulus unambiguously related to a season (e.g. ‘bikini’ for summer) and presented with another object name either belonging to another season (e.g. ‘coat’ for winter) or with a ‘neutral’ object, i.e. not related to any season (e.g. ‘car’). All the experimental stimuli had been previously piloted in a study with different participants.

### Procedure

The 3 different tasks (quantity, non-quantity categorical and perceptual-decision) with 2 stimuli (numbers and object names) were blocked (6 stimuli per block) and presented in pseudo-random order. Participants viewed pairs of stimuli presented one above the other with a fixation cross in the middle of the computer screen.

**Fig. 1:**
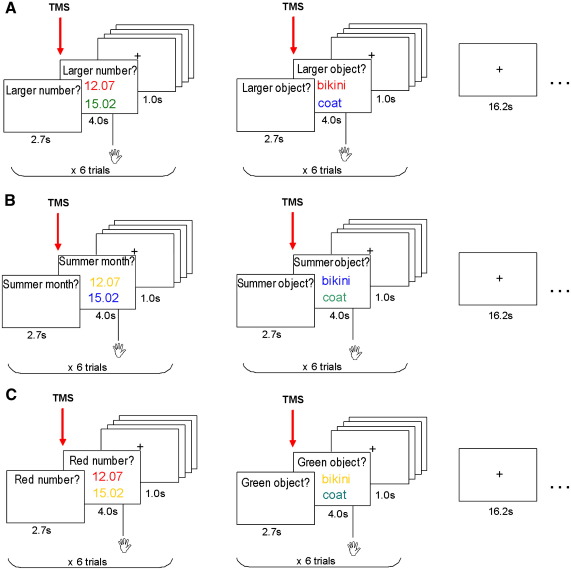
Experimental design. In each trial, participants viewed pairs of stimuli presented one above the other with a fixation cross in the middle of the computer screen. Stimuli could be either two Arabic numbers (left column) or two object names (right column) presented in one of four possible colours (red, yellow, blue, green). Participants were instructed to indicate with a button press which of these stimuli was the correct response to a question consisting of two key words presented above the upper stimulus before and during the stimulus display. There were three types of tasks: for quantity tasks (A), there were four possible types of questions: ‘larger?’, ‘smaller?’, ‘more?’, ‘less?’. For non-quantity categorical tasks (B), there were four different types of questions: ‘summer month/object?’, ‘winter month/object?’, ‘sleeping time/object?’, ‘working time/object?’ (either ‘month’ or ‘object’ was displayed depending on the stimulus condition). For the stimulus-colour decision (C) the questions were: ‘blue/red/yellow/green number or object? The 3 different tasks (quantity, non-quantity categorical and colour-decision) with both stimuli (numbers and object names) were blocked (6 stimuli per block) and presented in pseudo-random. Each condition (e.g. quantity) was first presented with numerical stimuli, e.g. ‘larger number?’ (or object names, e.g. ‘larger object?’) and followed by another block with object names (or numerical stimuli) in a counterbalanced order. Presentation of blocks of the same task with both stimuli was followed by about 16-second rest period where subjects were asked to maintain fixation on a cross in the middle of the computer screen. Trials where the correct answer was the upper or the lower stimulus were presented in equal proportion. (For interpretation of the references to colour in this figure legend, the reader is referred to the web version of this article.)

For each pair, they were instructed to indicate with a button press which stimulus was the correct response to a question consisting of two key words presented above the upper stimulus before and during a block of 6 trials (see Fig. 1). On every trial, participants were instructed to press the upper or the lower-arrow keys on the keyboard for the upper or the lower stimulus respectively.^3^ Trials where the correct answer was the upper or the lower stimulus were presented in equal proportion.

In each pair, each stimulus was presented in one of four possible colours (red, yellow, blue, green). In the quantity and in the non-quantity categorical tasks, subjects were instructed to ignore the colour of the stimuli, and to focus on the question presented above the stimuli. In the perceptual task (colour-decision) they were asked to choose the stimulus whose colour corresponded to the colour indicated by the question above the stimuli. Subjects were instructed to select the stimulus according to the colour of the ink and not according to the colour of the object (e.g. they should not select red just because the object name was for example ‘strawberries’ or ‘tomatoes’).

Participants were told that the number stimuli could indicate either: i) quantities, ii) dates, or iii) times. The instructions for the number stimuli were as follows: For the larger/smaller and more/less questions, participants were told that numbers referred to an amount and that they should choose the larger (or smaller) number in each pair irrespective of the wording of the question (i.e. “larger” or “more”). For summer/winter questions, participants were told that each number indicated either a summer or a winter month in the Northern hemisphere (all participants were British and raised in the UK). They were told that summer months were ‘June’, ‘July’, and ‘August’ and winter moths were ‘December’, ‘January’ and ‘February’ and that these months followed a day (1–31) separated with a dot (12.07) rather than the more familiar slash (12/07). They were instructed to select either the summer or the winter month in each pair of stimuli depending on the question. For the working/sleeping questions, participants were told that working or sleeping times were in terms of a 24-hour clock; and that working times were between 8am and 6pm, and sleeping times were between 10pm and 7am. Participants were discouraged from considering jobs that include night shifts.

For object names, the instructions were the same as those for the numbers except that the processing required for “more/less” questions was not the same as that required for “larger/smaller” questions. Instead, during the more/less questions participants were instructed to select the stimulus that was more (or less) numerous than the other, for example ‘socks vs thermos’, ‘stars vs moon’, ‘bed vs blanket’, ‘deck chair vs swimming pool’, ‘snowflakes vs snowman’ or ‘cherries vs melon’. Prior to the experiment, participants underwent a practice session in order to familiarize themselves with the task procedure.

### TMS design

TMS was delivered over two brain sites and sham stimulation was also given over the same regions. TMS was applied using a Magstim Rapid Rate stimulator (Magstim Company Ltd., UK) and a focal 8-shaped coil with each wing measuring 70 mm in diameter. TMS was applied at 60% of the maximum output of the stimulator machine at 10 Hz frequency for 500 ms at the stimulus onset. A fixed level of stimulation was used as it has been shown that there is little correlation of TMS stimulation thresholds between different brain areas (Stewart et al., 2001). Sham stimulation was produced by orienting the coil sideways, so that the sound and feeling of physical contact with the head were similar to those produced by real stimulation. For TMS, the stimulation coil rested tangentially on the subject's scalp and the handle pointing posteriorly parallel to the subject's midsagittal plane as calculated by frameless stereotaxy (Paus and Wolforth, 1998). Target regions were identified by a previous fMRI study run with the identical experimental design on different subjects^4^ (Cappelletti et al., in press) and consisted of two regions on the left and right IPS (MNI coordinates: − 42, − 40, 42 and 38, − 44, 40 respectively). These areas corresponded to the brain regions commonly activated by numbers and object names and identified by the main effect of conceptual tasks (over quantity and non-quantity for numbers and object names) relative to fixation. To ensure that these effects were not driven by one condition only, we used the inclusive masking option in SPM to identify the main effect of conceptual tasks relative to fixation in areas that were activated by both (i) conceptual tasks on numbers and (ii) conceptual tasks on object names at *p* < 0.01. To control for any correlation between conditions, a correction was made for non-sphericity using standard SPM5 procedures.

In order to locate the site of stimulation accurately, we used a frameless stereotactic system (Brainsight software, Rogue Research, Montreal, Canada). This system allows the precise localization of anatomical areas using the MRI images of the participants (e.g. Dorward et al., 1999), and has been successfully used in previous fMRI-guided TMS studies (e.g. Devlin et al., 2003). Prior to the experiment, a high-resolution T_1_-weighted MRI scan of the brain of each TMS participant was obtained in order to locate the two target regions relative to external landmarks on the head. These landmarks consisted of the bridge and tip of the nose and the tragus of the ears that are visible on both the subject's MRI scan and on his/her head. The 3D location of these landmarks was registered using an optical tracking system. This system uses an infrared camera that can detect reflectors attached to the objects of interest (i.e. the coil and the subject's head, Paus, 1999). The individual Brainsight localizations can be seen in Supplementary Material.

**Fig. 2:**
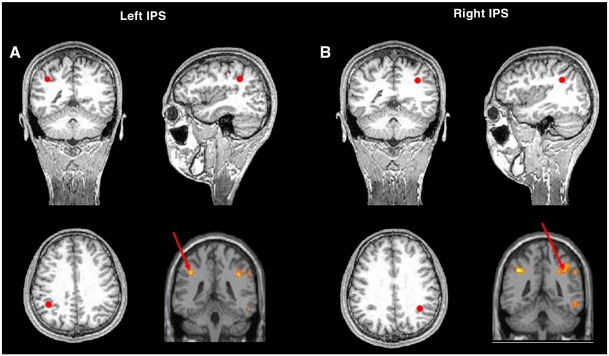
Location of the TMS target areas based on fMRI coordinates. The TMS target areas in the (A) left and (B) right IPS on the sagittal, coronal and axial view of the brain of a representative participant (anti-clock wise from top right). Target regions were identified by a previous fMRI study run with the identical experimental design on different subjects (Cappelletti et al., under review) and consisted of two regions on the left and right IPS (bottom right of A and B). These areas corresponded to the brain regions commonly activated by numbers and object names and identified by the main effect of conceptual tasks (over quantity and non-quantity categorical for numbers and object names) relative to fixation. Precise location of TMS target regions was obtained using a frameless stereotactic system (Brainsight software, Rogue Research, Montreal, Canada).

Stimulation locations were calculated for each subject individually using their structural scan and the coordinates obtained from the fMRI analysis, similar to other TMS studies (e.g. Kalla et al., 2008; Sack et al., 2009). Briefly, this involved normalisation of the structural scan against a standard template (using the FSL software package, FMRIB, Oxford, UK) which produced both a normalised structural scan and a mathematical description of the applied transformation. This transformation was then reverse applied to the coordinates to be targeted, giving their location in untransformed space. These were marked on the scan in Brainsight, and the scalp locations overlying each served as the targets for TMS. The precise localization of the target regions of a representative participant as well as the results of the fMRI group analysis is shown in Fig. 2.

For each area stimulated there were nine blocks, three for each experimental condition (TMS, sham and no-stimulation respectively). Each block consisted of 18 trials for each task (quantity, non-quantity and colour decision) for each stimulus (numbers or object names); therefore each subject performed a total of 54 trials per task per stimulus in each experimental condition for a total of 324 trials for each experimental condition. The order of the experimental conditions and of the block pairs (i.e. each task with both stimuli) was presented in pseudo-random order across subjects to avoid learning and practice effects.

### Participants

Six neurologically normal and native-English participants gave their informed consent to participate in the TMS study (3 males, mean age 22.2, range 21–23). The study had been approved by the Ethics Committee of the Institute of Neurology in London and was performed in adherence with TMS safety guidelines (Wasserman, 1998).

### Analysis of TMS data

The proportion of errors and the mean reaction times (RTs) were calculated for each subject in each condition. Response times below 200 ms (i.e. anticipatory responses) and above 2 standard deviations of the overall mean of each individual (i.e. delayed responses) were excluded from the data set following a procedure which is common practice in data analysis and in previous TMS studies (e.g. Martin et al., 2004; Naeser et al., 2005). Furthermore, using this approach, we decreased the likelihood that our results were driven by outliers as we used tests that assume normal data distribution. By using the above 2SD criterion, 3.8% of the total number of responses were outliers and were therefore excluded from the analysis. Bartlett's test was used to check for homogeneity of variance and no data transformation was necessary.

A *t*-test compared the sham and the non-stimulation conditions across tasks to test whether there was any difference between them. As no difference emerged (see analysis below), we compared the TMS condition to sham rather than no-stimulation as sham is the most similar to TMS in terms of the sound produced and physical contact with the head.

A 4 × 2 × 3 analysis of variance (ANOVA) with condition [left and right TMS-IPS and left and right sham-IPS], stimulus-type [numbers and object names] and task [quantity, non-quantity categorical and colour-decision] as within-subject factors was conducted on RTs of correct answers. The analysis aimed at testing for stimulation effects and at verifying whether these effects interacted with task and/or stimulus. In addition, two-way ANOVAs were performed on RTs of correct answers for each task with condition [left and right TMS-IPS and sham] and stimulus-type [numbers and object names] as factors. We also tested whether TMS differentially affected numbers that were close vs far apart, i.e. ‘distance effect’. In the quantity tasks with numbers, the distance between 2 numbers was calculated by subtracting one number from the other. In contrast, in the non-quantity tasks with numbers, rather than the ‘internal distance’ between the 2 numbers, we calculated the distance between each numerical stimulus in the pair and the next standard and we then averaged these values for the numerical stimuli in each pair. Reported results were corrected for non-sphericity using Greenhouse–Geisser correction.

When appropriate, we performed post-hoc paired comparisons using student *t*-test and used Fisher LSD correction for multiple comparisons. Statistical significance refers to a two-tailed *p* value < 0.05.

## Results

TMS did not affect the participants' accuracy in any of the tasks [overall 4.6% of errors, no significant difference between TMS and sham across tasks and stimuli, *p* > 0.1]. This is consistent with similar paradigms or tasks used in posterior parietal cortex TMS showing that tasks performed at high levels of accuracy are likely to result in RT deficit rather than increase in error rate following TMS (Alexander et al., 2005; Ashbridge et al., 1997; Cappelletti et al., 2007).

There was no significant difference between sham and no-stimulation across any of the tasks or stimuli used [*t*(5) = 0.45, *p* = 0.67, n.s.].

The three-way ANOVA revealed a significant main effect of task [*F*(2,10) = 41.74, *p* < 0.001]. Moreover, the interaction of stimulus-type and task [*F*(2,10) = 9.36, *p* < 0.005], and the three-way interaction of condition, stimulus-type and task [*F*(6,30) = 2.36, *p* < 0.05] were all significant.

### Quantity task

**Fig. 3:**
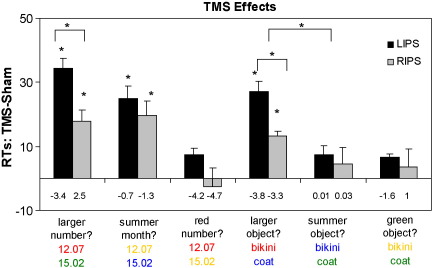
Performance impairments following TMS. Bars indicate the mean difference in response time in ms in the left (black bars) and right IPS (grey bars) after TMS relative to sham stimulation to the same areas, for quantity, non-quantity categorical and colour decisions with numbers and object names respectively. TMS conditions in which response times significantly differed from sham stimulation or between conditions are indicated by an asterisk above the bars. Numbers below bars indicate the mean differences in error rates between TMS and Sham in each experimental condition.

A two-way ANOVA revealed a significant interaction of condition (left and right TMS and sham) and stimulus-type (numbers and object names), [*F*(2,10) = 9.31, *p* < 0.006]. Specifically, performance in quantity tasks with numbers (e.g. which is larger: 12.07 or 15.02?) was significantly impaired (slower performance) by stimulation over the IPS relative to sham stimulation of the same areas, with longer RTs following rTMS to either left [871.63 ms, 34.5 ms difference with sham, *t*(5) = 9.56, *p* < 0.001] or right IPS [855.56 ms, 17.8 ms difference with sham, *t*(5) = 4.91, *p* = 0.004]. Left and right IPS stimulation differed significantly, left IPS-TMS inducing a larger impairment than right IPS-TMS [*t*(5) = 3.25, *p* < 0.02] (See Fig. 3). Moreover, left and right IPS-TMS impairment was greater for comparisons of close numbers relative to numbers far apart, i.e. distance effect [left IPS: *t*(5) = 2.593, *p* < 0.04 right IPS *t*(5) = 11.14, *p* < 0.001]. A distance effect was also observed in both the sham [*t*(5) = 2.76, *p* < 0.03] and the non-TMS conditions [*t*(5) = 10.99, *p* < 0.001].

Similarly, performance in quantity tasks with object names (e.g. larger object: ‘bikini’ or ‘coat’?) was significantly delayed after stimulation over the left [1083.2 ms, 27.03 ms difference with sham, *t*(5) = 8.38, *p* < 0.001] or right IPS [1069.86 ms, 13.3 ms difference with sham, *t*(5) = 9.56, *p* = 0.001] compared to sham stimulation over the same areas. Left and right IPS stimulation differed significantly, left IPS-TMS again inducing a larger impairment than the right IPS-TMS [*t*(5) = 3.49, *p* < 0.017].

### Non-quantity categorical task

A two-way ANOVA revealed a significant interaction of condition and stimulus-type [*F*(2,10) = 7.28, *p* < 0.01]. In particular, performance in the non-quantity categorical task with numerical stimuli (e.g. summer date: ‘23.07’ or ‘15.02’?) was significantly delayed following stimulation to the left [1118.57 ms, 24.9 ms difference with sham, *t*(5) = 6.42, *p* < 0.001] or right IPS [1113.44 ms, 19.7 ms difference with sham, *t*(5) = 4.5, *p* = 0.006] compared to sham stimulation of the same areas. Unlike the quantity task, there was no significant difference between left and right IPS stimulation [*t*(5) = 0.76, *p* = 0.5].

Left and right IPS-TMS impairment was greater for comparisons of close numbers when they referred to times, e.g. working time: ‘23.07’ or ‘15.02’? [left IPS: *t*(5) = 16.7, *p* < 0.001; right IPS *t*(5) = 6.5, *p* < 0.001], but not when they referred to dates, e.g. summer month: ‘23.07’ or ‘15.02’? [left IPS: *t*(5) = 0.61, *p* = 0.56, n.s.; right IPS *t*(5) = 2.12, *p* = 0.87, n.s.]. Similarly, a distance effect was also observed in both the sham [*t*(5) = 5.7, *p* < 0.002] and the non-TMS conditions [*t*(5) = 11.94, *p* < 0.001] for numbers referring to time but not to dates [*t*(5) = 0.8, *p* = 0.4 and *t*(5) = 0.38, *p* = 0.72 for sham and non-TMS respectively].

Relative to sham, performance following IPS-TMS in the non-quantity categorical task with object names (e.g. summer object: ‘bikini’ or ‘coat’?) remained unchanged following stimulation to the left [926.5 ms, 7.37 ms difference with sham, *t*(5) = 2.53, *p* = 0.06] and right IPS [929.4 ms, 4.4 ms difference with sham, *t*(5) = 8.3, *p* = 0.45]. There was no significant difference between left and right IPS-TMS stimulation [*t*(5) = 0.07, *p* < 0.95].

### Perceptual (colour-decision) task

A two-way ANOVA showed a non-significant interaction of condition and stimulus-type [*F*(2,10) = 3.53, *p* < 0.07].

## Discussion

This study investigated the role of the left and right IPS regions in processing quantity with numerical and non-numerical stimuli, and in conceptual tasks with numbers requiring or not quantity manipulation. We showed that quantity judgments with numerical and non-numerical stimuli (e.g. choosing the larger of ‘12.07’ and ‘15.02’ or the larger in size of ‘bikini’ and ‘coat’) were equally affected following IPS-TMS. Moreover, number conceptual processing either involving or not involving quantity manipulation (e.g. choosing the larger of ‘12.07’ and ‘15.02’, or attributing a summer date to either ‘12.07’ or ‘15.02’) was also equally impaired following IPS-TMS. Our results also showed a distance effect in both quantity and non-quantity categorical judgments. This is consistent with previous results indicating that distance effect can be observed not just with any continuous quantity dimension such as size of symbols (e.g. Cohen Kadosh et al., 2005; Kaufmann et al., 2005; Pinel et al., 2004; Tang et al., 2006) and luminance (Cohen Kadosh et al., 2005; Pinel et al., 2004), but also with non-quantity dimensions such as letters of the alphabet (e.g. Fulbright et al., 2003) or social status (Chiao et al., 2004). It may be suggested that the distance effect induced by IPS-TMS is simply due to TMS affecting the most difficult conditions, i.e. close vs distant numbers, rather than interfering with numerical processing *per se*. We note, however, that if this were the case TMS-induced distance effects should have been observed in all numerical tasks. In contrast, we showed that in the non-quantity categorical tasks with numbers (summer/winter month; working/sleeping time), the distance effect was affected by TMS in one of these tasks (working/sleeping time) but not the other (summer/winter month), although they were the same in terms of difficulty at baseline. Some previous studies have already shown that IPS-TMS may induce different degrees of interference on the distance effect in numerical task of the same difficulty level at baseline (e.g. Cappelletti et al., 2007).

These findings challenge past results by showing that the role of IPS in quantity processing is stimulus-independent and its role in numerical cognition is not specific for the conceptual task performed with numbers. Two further critical conditions revealed the true specificity of the IPS contribution to numerical processing: IPS-TMS showed no impairment for perceptual decisions on numerical stimuli in the absence of any conceptual task (i.e. colour judgment), nor for conceptual decisions that did not involve quantity or number stimuli (e.g. choosing a summer object between ‘bikini’ and ‘coat’). From this, we suggest that the simple presence of numbers is not sufficient for the IPS to be critically involved, but conceptual-level operations are also necessary. However, conceptual operations on their own are also not sufficient to engage the IPS, but must be specifically oriented to numerical concepts.

### One or more quantity mechanisms?

The issue of whether the quantity expressed by different stimuli is processed in the same way or via distinct mechanisms is a long standing issue. The seminal work of Moyer and Landauer (1967) suggesting that the magnitude of all stimuli is processed in the same way has received support at the theoretical level (e.g. Campbell, 1994; Dehaene, 1997; McCloskey, 1992; Noel and Seron, 1993), and more recently has motivated research into the neuronal correlates of magnitude processing. These have been identified mainly in the parietal regions (Cappelletti et al., 2007, in press; Cohen Kadosh et al., 2005, 2007a,b; Dehaene et al., 2003; Pinel et al., 2001, 2004; Piazza et al., 2004, 2007), and a recent theoretical proposal has suggested these areas as the locus of stimulus-independent magnitude processing according to a common metric (Walsh, 2003). However, dissociations between quantity processing of Arabic numbers and of other magnitude dimensions such as the size of physical stimuli have been reported in neuropsychological patients (e.g. Cipolotti et al., 1991; Lemer et al., 2003) and not all functional imaging studies have shown consistent results as to whether the bilateral IPS is involved in stimulus-independent magnitude processing (e.g. Cohen Kadosh et al. 2007a,b; Pinel et al., 2004) or whether the left or the right IPS is differentially involved (e.g. Castelli et al., 2006; Cohen Kadosh et al., 2007a,b; Pinel et al., 2004 for right IPS activation; Fias et al., 2003; Cohen Kadosh et al., 2005 for left IPS activation). One factor that may account for these discrepancies is the way in which magnitudes change — luminance is continuous, for example, while number changes are discrete (e.g. Cohen Kadosh et al., 2007a,b; Pinel et al., 2004) irrespective of the underlying representation. Another factor, as formalised by Stevens (1975), is the way in which magnitude changes are experienced — weight differences are experienced as “more than” or “less than” another weight, while frequency and saturation changes modify the categorical perception of pitch and colour respectively. A third reason for the discrepancy of previous results is the possible confound between numerical processing and response selection, both engaging the parietal lobes (e.g. Cappelletti et al., in press; Goebel et al., 2005). However, it was beyond the purpose of this study to address this issue. A fourth difference is that numerical magnitude judgments (i.e. is 65 bigger than 55?) may necessitate the retrieval of learnt information while physical size and luminance decisions may be based on visual stimulus properties rather than memory-related strategies. Our study avoided this confound by using magnitude dimensions with similar processing requirements: choosing the larger of two Arabic numbers (e.g. ‘12.07’ vs ‘15.02’) or of two objects (e.g. ‘bikini’ vs ‘coat’) relies in both cases on the retrieval of associated information which is not contained in the stimuli. By using this novel experimental manipulation we have provided new evidence suggesting that the bilateral IPS is necessary for processing magnitude expressed by different stimuli when these are based on similar cognitive resources.

### Quantity and non-quantity conceptual number processing in the IPS

We have also shown that the IPS regions are equally involved in quantity processing and in other conceptual tasks with numbers that do not require quantity manipulation, i.e. categorical tasks. The idea that number semantics can be extended to include non-quantity conceptual operations on numbers has been proposed by some previous theoretical accounts (Butterworth, 1999; Dehaene, 1997). However, this proposal has not yet been systematically investigated as most of the previous studies focused on quantity processing. By showing that the parietal regions are involved not only in processing the quantitative features of numbers but also in other non-quantitative conceptual manipulations our results support the idea that number semantics in the IPS is not limited to the processing of magnitude but can be extended to include other numerical conceptual processing (Butterworth, 1999; Dehaene, 1997).

Our findings show that the involvement of the IPS regions in number processing is modulated by the nature of the number task performed: responses were delayed only when subjects were performing quantitative and non-quantitative conceptual judgments but not perceptual judgments with numerical stimuli. These results are consistent with proposals that the parietal areas are engaged in the conceptual representation of numbers (Dehaene, 1997; Dehaene and Cohen, 1995), and with a recent fMRI study using the same experimental design showing the involvement of the IPS only in conceptual (quantity and non-quantity) but not in perceptual number tasks (Cappelletti et al., in press). How do these results combine with the most common view that number processing is so automatic that the simple presentation of numbers activates a sense of magnitude (e.g. Ansari et al., 2006; Dehaene and Akhavein, 1995; Eger et al., 2003; Thioux et al., 2005)? One possibility is that a magnitude representation might have been automatically activated in the colour task but TMS had no effect on colour processing, therefore no disruption by TMS was seen in that task. Another possibility is that different types of numerical representations may be triggered by different types of task requirements (Fischer and Rottmann, 2005; Shaki and Petrusic, 2005). In our study these numerical representations may differ in the conceptual (quantity and non-quantity) relative to perceptual tasks. Moreover, these representations may rely on different brain regions or may pose different task requirements on the same regions, therefore resulting in different TMS effects. As such, our results suggest a refinement of the idea of task-independent automatic magnitude activation and are in keeping with proposals suggesting that magnitude representations may interact with task requirements.

It still remains to be explained why some previous imaging studies showed similar parietal activation in numerical tasks whether they required quantity processing or not (e.g. Göbel et al., 2005; Eger et al., 2003; Shuman and Kanwisher, 2004; Tang et al., 2006; Thioux et al., 2005). For instance, Thioux et al. (2005) found no difference in IPS activation when contrasting number comparison to a task where subjects have to decide if numbers were written in plain characters or not. Similarly, Eger et al. (2003) reported IPS activation in a simple number detection task (i.e. indicate when a stimulus is a number) not requiring quantity manipulation. The simplest possibility is that not all activations reported in fMRI studies are indicative of an area being necessary for normal performance of that task. There are two reasons for this: first there is some 'redundancy' in perceptual and cognitive systems (e.g. Price and Friston, 2002); second, fMRI activations are the result of a particular subtraction and thus indicate activity elicited by one kind of stimulus/task *relative* to another stimulus/task. Necessity of an area to those stimuli/tasks can only be ascertained by interference. Another reason may lie in the type of tasks used or the response selection demands of the task. For instance, tasks such as stimulus detection (Eger et al., 2003) activate the parietal lobes because they require conceptual processing in the form of identity recognition (i.e. when distinguishing a number from another stimulus). Moreover, the perceptual tasks used in some of these studies (e.g. Göbel et al., 2005; Tang et al., 2006; Thioux et al., 2005) involved visual search processes that have been shown to activate the superior parietal lobes (e.g. Coull et al., 2003; Pollmann et al., 2003) whereas a colour detection task placed minimal demands on visuo-spatial mechanisms because it could be based on any unit of the string. Our findings that the left and right IPS are critically involved in conceptual but not perceptual processing of numbers is also in keeping with some previous studies based on fMRI adaptation paradigms (fMRIA, e.g. Cohen Kadosh et al., 2007a,b; Cantlon et al., 2006; Piazza et al., 2007). These studies showed that IPS adaptation (i.e. in the absence of any explicit task) is modulated by numerical magnitude but not by changes in the colour of numerical stimuli. Our results further expand this evidence by showing that the IPS plays a critical role in number processing and that this is specific for conceptual (quantity and non-quantity) but not perceptual number processing.

In conclusion, we have shown that the role of the IPS in numerical cognition is dependent on a combination of stimuli and task operations. Specifically, the left and right IPS are critical for: (1) performing quantity and non-quantity conceptual operations with numbers; and for (2) quantity processing irrespective of the stimuli used, i.e. numbers or object names. However, this study shows that the IPS is not critically involved in perceptual decisions based on numbers or in conceptual tasks that do not involve quantity or numerical stimuli.

## Figures and Tables

**Supplementary Figure:**
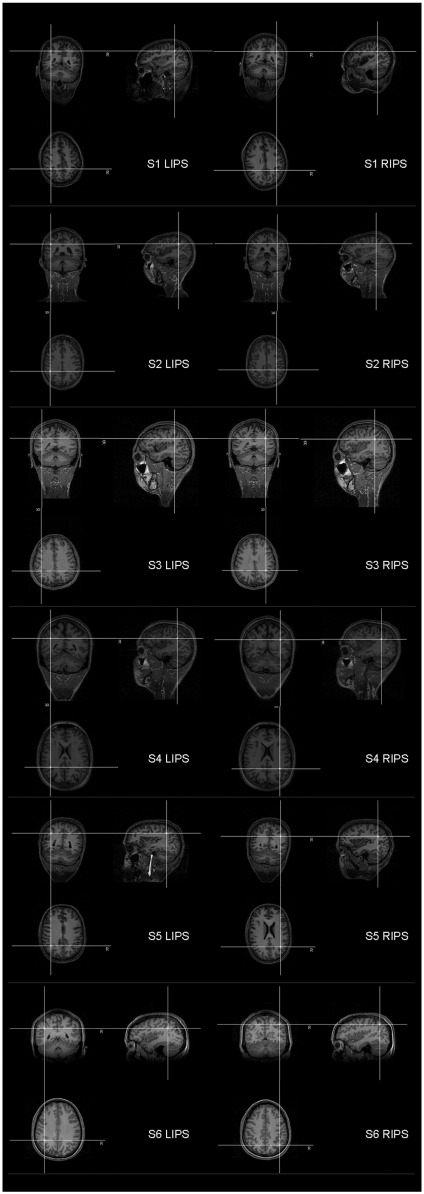


## Footnotes

1 In the text we refer to the intraparietal sulcus in terms of the brain regions around the sulcus, rather than the sulcus *per se*, although for brevity we indicate these as IPS or IPS regions.

2 The horizontal segment of the IPS is the middle part of the sulcus in the dorsal parietal lobe that intersects the postcentral sulcus (Ono et al., 1990).

3 Participants' fingers rested on the two designated keys of the keyboard for the whole duration of the experiment following a procedure common in two-choice response experiments. This is unlikely to result in response priming as correct responses were randomized.

4 Functional image analysis was performed using Statistical Parametric Mapping software (SPM5 software, Wellcome Trust Centre for Neuroimaging, London; http//www.fil.ion.ucl.ac.uk/spm). Scans were realigned, unwarped and spatially normalized (Friston et al., 1995) to the Montreal Neurological Institute (MNI) standard space. Functional images were then smoothed in the spatial domain with a Gaussian kernel of 6 mm FWHM to improve the signal to noise ratio. A high pass filter was used with a cutoff period of 128s.
